# Multiparametric Assays Capture Sex- and Environment-Dependent Modifiers of Behavioral Phenotypes in Autism Mouse Models

**DOI:** 10.1016/j.bpsgos.2024.100366

**Published:** 2024-07-20

**Authors:** Lucas Wahl, Arun Karim, Amy R. Hassett, Max van der Doe, Stephanie Dijkhuizen, Aleksandra Badura

**Affiliations:** Department of Neuroscience, Erasmus MC, Rotterdam, the Netherlands

**Keywords:** Animal behavior, Autism, Environmental effects, Heterogeneity, Mice, Sex differences

## Abstract

**Background:**

Current phenotyping approaches for murine autism models often focus on one selected behavioral feature, making the translation onto a spectrum of autistic characteristics in humans challenging. Furthermore, sex and environmental factors are rarely considered. Here, we aimed to capture the full spectrum of behavioral manifestations in 3 autism mouse models to develop a “behavioral fingerprint” that takes environmental and sex influences under consideration.

**Methods:**

To this end, we employed a wide range of classical standardized behavioral tests and 2 multiparametric behavioral assays—the Live Mouse Tracker and Motion Sequencing—on male and female *Shank2*, *Tsc1*, and Purkinje cell–specific *Tsc1* mutant mice raised in standard or enriched environments. Our aim was to integrate our high dimensional data into one single platform to classify differences in all experimental groups along dimensions with maximum discriminative power.

**Results:**

Multiparametric behavioral assays enabled a more accurate classification of experimental groups than classical tests, and dimensionality reduction analysis demonstrated significant additional gains in classification accuracy, highlighting the presence of sex, environmental, and genotype differences in our experimental groups.

**Conclusions:**

Together, our results provide a complete phenotypic description of all tested groups, suggesting that multiparametric assays can capture the entire spectrum of the heterogeneous phenotype in autism mouse models.

## Plain Language Summary

Traditional methods of studying behavior in mouse models of human disorders often focus on single behavioral features. The current study sought to address this gap by examining 3 autism mouse models, using a variety of traditional and novel behavioral tests and computational dimensionality reduction analyses, to create a “behavioral fingerprint” for each animal. This study also considered the effects of the environmental factor of housing conditions and sex on behavior in these models. The findings suggest that complex, multiparametric assays offer a more nuanced portrayal of behavioral variability in autism mouse models, showcasing the complex interplay of genetics, environment, and behavior.

Mouse models serve as invaluable tools in the comprehensive exploration of human neurodevelopmental disorders to understand the underlying pathophysiology, evaluate new mechanistic targets, and screen novel drugs (1, 2, 3, 4, 5). However, one of the limiting factors of using mouse models for complex disorders relates to their pronounced levels of phenotypic variability. This is difficult to study in the laboratory setting, which frequently resorts to tests that measure a limited number of behaviors (6).

Autism is a prime example of such a complex neurodevelopmental disorder with a broad spectrum of behavioral manifestations (7). This heterogeneity is in part a consequence of autism etiology, which not only has a large genetic component (8,9) but also is sensitive toward environmental modifiers (10). Therefore, autism diagnosis in humans is a lengthy process that encompasses direct observation of the individuals, which can be extended with neuropsychological evaluation tests, and interviews with parents or caregivers (11,12). There have recently been considerable scientific efforts to identify distinct subgroups within the spectrum of autism (13, 14, 15).

Preclinically, laboratory animal models have been used to establish autism-like phenotypes primarily through the development of mouse models with high-risk genetic mutations (2,16,17). Over the years, several behavioral paradigms have been developed, which have been commonly used to assess core autism deficits (18). These classical behavioral assays were mostly designed for single-purpose quantification of specific behavioral impairments (such as perseveration, repetitive behaviors, or social deficits). Recent computational advances have allowed exploration of behavioral domains in significantly greater detail, enabling classification of behaviors in home cages (19) and providing automated quantification of complex social behaviors (20, 21, 22). Additionally, methods that rely on unsupervised machine learning have provided tools to quantify so-called behavioral syllables, which have been defined as subsecond building blocks of more complex behaviors (23,24). These tools have recently been introduced for the quantification of diverse deficits in mouse models of neurodevelopmental disorders (25,26).

Research using animal models of autism has focused mainly on the genetic mechanisms underlying this disorder. Several studies investigated home-cage enrichment as an environmental modifier, which has been shown to ameliorate the autism-like phenotype in some genetic models (27, 28, 29). Furthermore, sex is also an important factor of autism risk, with male to female ratio estimates ranging from 4:1 (30,31) to 2:1 (32). Autism in females often presents differently from the stereotype of autistic behavior (33,34) and is therefore frequently mis- or underdiagnosed (35,36). Similar sex bias is present in fundamental autism research using mouse models, where males are overrepresented (37). Therefore, it is increasingly urgent to establish an autism phenotype in female mice representing the most commonly used genetic autism models, while taking environmental modifiers into consideration.

Here, we combined phenotyping using classical behavioral assays with recent advances in automated classification of individual and group behaviors to develop a new dimensionality reduction approach that captured sex- and environment-dependent variability of behavioral phenotypes in *Shank2*^*−/−*^ and *Tsc1*^*+/−*^ autism mouse models as well as Purkinje cell–specific, heterozygous *Tsc1* mutant mice (further referred to as *L7-Tsc1*^*flox/+*^ mice). *SHANK* genes, which play an essential role in the formation and the maintenance of synapses, have an established association with autism as monogenic risk factors (38). We have used homozygous *Shank2*^*−/−*^ mutant mice, as to date, publications on *Shank2* mice have largely focused on homozygotic deletions (39, 40, 41, 42). Loss-of-function mutations in the *TSC1* gene result in tuberous sclerosis complex, a genetic disorder with high levels of comorbidity for autism (43). Additionally, we used Purkinje cell–specific mutants of *Tsc1* because cerebellar injury at birth has been described as one of the risk factors for autism in preterm infant cohorts (44), and in the past few years, there has been an increased interest in using cerebellar-specific mouse models in autism research (17). Purkinje cell–specific deletion of the *Tsc1* gene has been shown to lead to autism-like deficits (45,46) and has been used to test pharmacological interventions (47,48).

Our approach can be applied to a range of behavioral data outputs and neurodevelopmental and neuropsychiatric animal models to show meaningful lower-dimensional behavioral dynamics that allow quantitative description of heterogeneous mouse behavior.

## Methods and Materials

### Experimental Procedures

All experimental animal procedures were approved a priori by an independent animal ethical committee (DEC-Consult, Soest, the Netherlands), as required by Dutch law and conform to the relevant institutional regulations of the Erasmus Medical Center and Dutch legislation on animal experimentation (CCD approval: AVD1010020197846).

### Animals

We used a total of 325 mice, including 2 mouse lines with global gene deletion: *Shank2*^*−/−*^ (*n* = 59) (49) and *Tsc1*^*tm1Djk*^, referred to as *Tsc1*^*+/−*^ (*n* = 54) (50) lines and a Purkinje cell–specific *CrB6.Cg-Tg(Pcp2-cre)3555Jdhu/J::Tsc1*^*flox/+*^ line, referred to as *L7-Tsc1*^*flox/+*^ (*n* = 58) (45) (Figure S1), with their wild-type (WT) littermates (*n* = 60, *n* = 54, *n* = 51, respectively). Mice were weaned at postnatal day 21 and group housed (3 mice per cage, same sex) with mixed genotypes of the same genetic model, with food and water ad libitum, on a regular 12-hour light/dark cycle. Animals were raised in either standard housing (335 cm^2^) or environmentally enriched cages (800 cm^2^), both with wood chip bedding (Lignocel) and nesting material. In addition, environmentally enriched cages contained a running wheel, wooden blocks, plastic tubes, and paper houses. For details on genotyping, background, animal numbers, animal handling, and choice of housing 3 mice per cage, see Supplemental Materials and Methods and Figure S2.

### Order of Experiments

For all mice, the first week of the behavioral battery consisted of a Live Mouse Tracker (LMT) recording. For the second week, the following schedule was kept at all times: day 1, the social chamber test; day 2, elevated plus maze; day 3, open field test; day 4, marble burying test; and day 5, Motion Sequencing (MoSeq). In the last week of the behavioral battery, the water Y maze was performed.

### Single-Trait Behavioral Assays

Detailed description of experimental procedures regarding single-trait assays is available in the Supplement. In short, we used 1) a 3-chamber social preference test to quantify sociability (51), 2) an elevated plus maze to examine anxiety-like behavior (52), 3) an open field test to examine anxiety-like behavior and locomotor activity (53), 4) a marble burying test (54) to assess repetitive burying behaviors, and 5) a water Y maze paradigm to examine cognitive flexibility (26,55). Multivariate analysis of variance with post hoc testing has been used for statistical analysis, taking the interactions between environment, sex, and genotype under consideration.

### Multiparametric Assays

Multiparametric assays were performed to capture a wide range of behavioral profiles in an open field arena. We used the following 2 methods: the MoSeq (23) and the LMT (22). Animals were habituated to the testing room for 1 hour before experiments. Multivariate analysis of variance with post hoc testing has been used for statistical analysis of syllables in the MoSeq analysis and behaviors in the LMT analysis, taking the interactions between environment, sex, and genotype under consideration.

#### Motion Sequencing

MoSeq (version 1.1.1b0) was used as described previously (23,56) to classify behavioral syllables. MoSeq was performed in single mice in a 50 × 50 cm opaque acrylic open field cage placed inside a 120 cm wide, 120 cm deep, and 160 cm tall behavioral box for a duration of 60 minutes and recorded at 30 fps. Data were analyzed with existing MoSeq and custom scripts (https://github.com/BaduraLab/ASD-mouse-model-analysis). To ensure unbiased inclusion of all expressed behavioral syllables, the model was trained using the full dataset, thereby containing animals from all experimental conditions. For further analysis, we included 41 syllables with the highest usage over all mice to have a balanced input for dimensionality reduction analyses, accounting for 67% of the total frames in our dataset.

#### Live Mouse Tracker

The LMT (22) was used to track mice group behavior. One week before experiments, mice were surgically injected subcutaneously with radiofrequency identification tags (134 kHz glass probe ISO 11784/11785m; Priority1Design) into the flank region, under isoflurane anesthesia (induction: 4%V/V in O_2_, maintenance: 2%–2.5% V/V in O_2_; flow rate ∼0.5 L/min) and subcutaneous injection of Rimadyl (5 mg/kg). The LMT setup was built as previously described (22) and placed into the same box used for MoSeq experiments. LMT experiments were performed using the same cage configuration as the home cage, meaning that the same group of 3 mice that were housed together from weaning was placed and recorded in the LMT setup. Mice were recorded for a duration of 60 minutes. Data were analyzed with existing LMT scripts and custom analysis scripts (https://github.com/BaduraLab/ASD-mouse-model-analysis). The LMT software is able to track and analyze 33 behaviors in a group of 4 mice. Because we tested groups of 3 animals, the behaviors that describe 4 animals have been omitted. This resulted in 29 identifiable behaviors.

### Normalization

Data from single-trait behavioral assays were normalized to create radial plots showing an overview of the behavioral phenotype per autism mouse model. Detailed description of normalization procedure can be found in the Supplement.

### Dimensionality Reduction and Machine Learning Classification

The measurements from the single-trait behavioral assays and the multiparametric assays were gathered in a singular dataset. Dimensionality of the data was reduced using a 2-stage principal component analysis–linear discriminant analysis algorithm. We used SciKitLearn (version 0.24.2) python library (57) to train linear classifiers. After cross-validation, a linear support vector machine classification method was chosen. Coding was done in both MATLAB (R2022a; The MathWorks, Inc.) and Python (version 3.9.7). Detailed description of dimensionality reduction and machine learning classification methodology can be found in the Supplement.

## Results

### Classical Behavioral Assays Capture Modulating Effect of Environmental Enrichment on Behavioral Impairments in *Shank2*^*−/−*^ Mice

**Figure 1:**
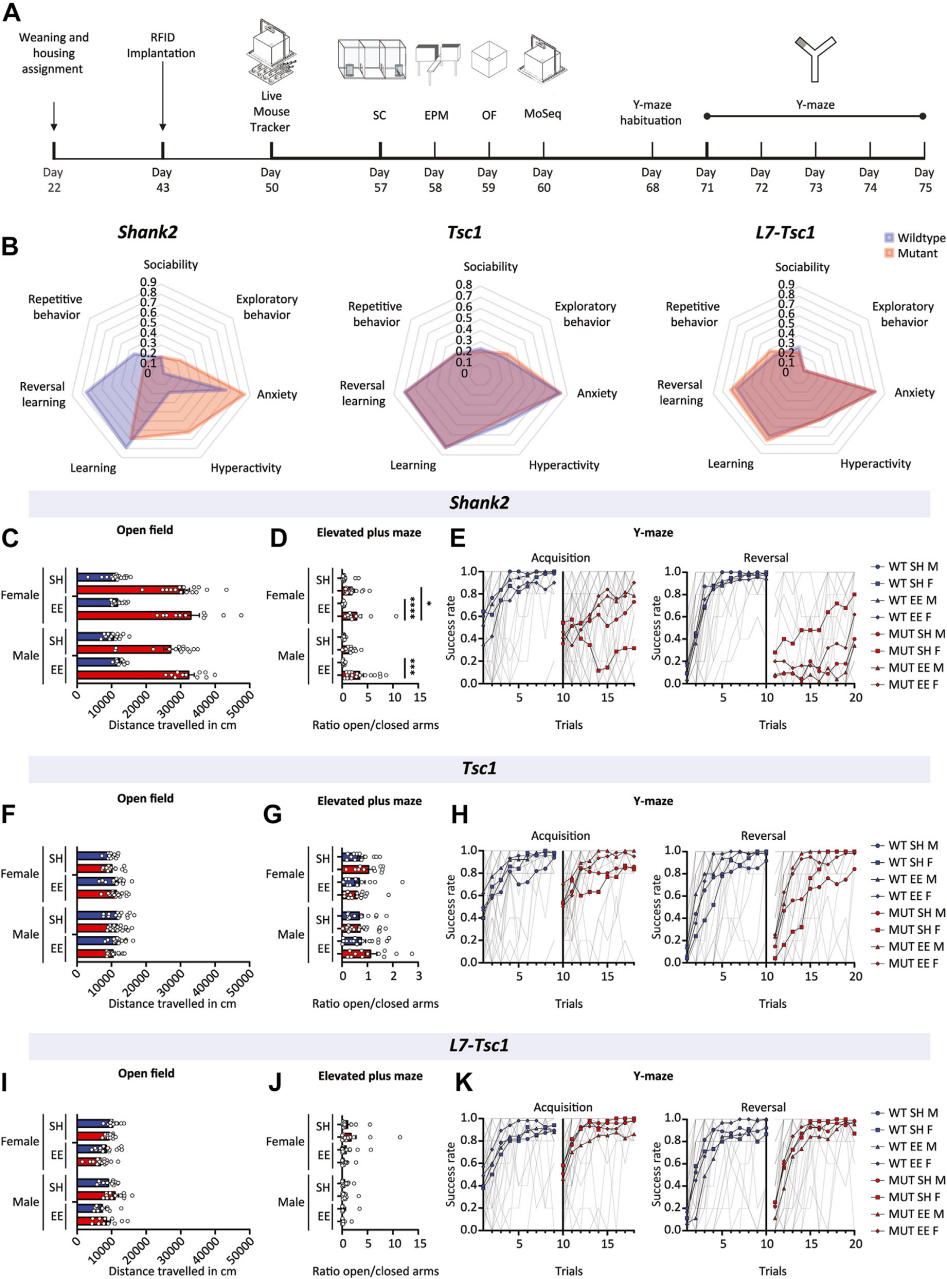
Behavioral phenotyping of autism mouse models using classical behavioral assays. **(A)** Experimental timeline of behavioral experiments. **(B)** Normalized scores in classical behavioral assays per mouse model of autism. **(C, F, I)** Distance traveled in the OF test. Data presented as mean with SEM (3-way analysis of variance with Holm-Šidák’s multiple comparisons test). **(D, G, J)** Ratio of the time spent in the open arms of an EPM divided by the time spent in the closed arms. Data presented as mean with SEM (3-way analysis of variance with Holm-Šidák’s multiple comparisons test). **(E, H, K)** Performance in the acquisition phase of the Y maze (left) and the reversal phase (right). Data presented as mean with SEM (1-way analysis of variance with Šidák’s multiple comparisons test). ∗*p* ≤ .05, ∗∗∗*p* ≤ .001, ∗∗∗∗*p* ≤ .0001. *Shank2*, *n* = 114; *Tsc1*, *n* = 108; *L7-Tsc1*, *n* = 105 mice. EE, environmentally enriched; EPM, elevated plus maze; F, female; M, male; MoSeq, motion sequencing; MUT, mutant; OF, open field; RFID, radiofrequency identification; SC, social chamber; SH, standard housing; WT, wild-type.

To test environmental effects and sexual behavioral dimorphism on a range of phenotypes, male and female mice were assigned to either standard or environmentally enriched housing at postnatal day 21 (Figure 1A). At 6 weeks old, animals were injected with radiofrequency identification tags to quantify group behaviors using the LMT (22). Next, all animals underwent a series of classical behavioral assays as well as MoSeq (23).

In the classical behavioral assays, *Shank2*^*−/−*^ mice were found to show behavioral impairments in anxiety-related behavior (Figure 1B and Figure S3A) and a pronounced hyperactivity phenotype (genotype: *p* < .0001) (Figure 1C) in the open field test and deficits in learning (*p* < .0001) and reversal learning (genotype: *p* < .0001) during the water Y maze compared with their WT littermates (Figure 1E). *Shank2*^*−/−*^ mice showed increased levels of exploratory behavior (genotype: *p* < .0001) (Figure 1D) in the elevated plus maze, an effect that was exacerbated in animals raised in enriched environments (genotype × housing: *p* = .0076). *Shank2*^*−/−*^ mice showed similar levels of social behavior compared with WT littermates and reduced burying behavior (genotype: *p* < .0033) (Figure S3B, C). Neither the *Tsc1*^*+/−*^ (Figure 1B, center) nor the heterozygous *L7-Tsc1*^*flox/+*^ (Figure 1B, right) mice showed significant impairments in the classical behavioral assays (Figure 1F–K and Figure S3).

### MoSeq Reveals Genotype-, Sex-, and Environment-Specific Behavioral Phenotypes in Mouse Models of Autism

Next, we performed MoSeq to study the underlying behavioral state of the animals, using unsupervised machine learning to find repeated behavioral patterns referred to as behavioral syllables (23). Here, we aimed to look at the innate behavioral differences expressed by the animals by studying the expression of behavioral patterns in an arena without external stimuli to see whether altered behavioral phenotypes of autism mouse models can be segmented during spontaneously expressed behavior.

**Figure 2:**
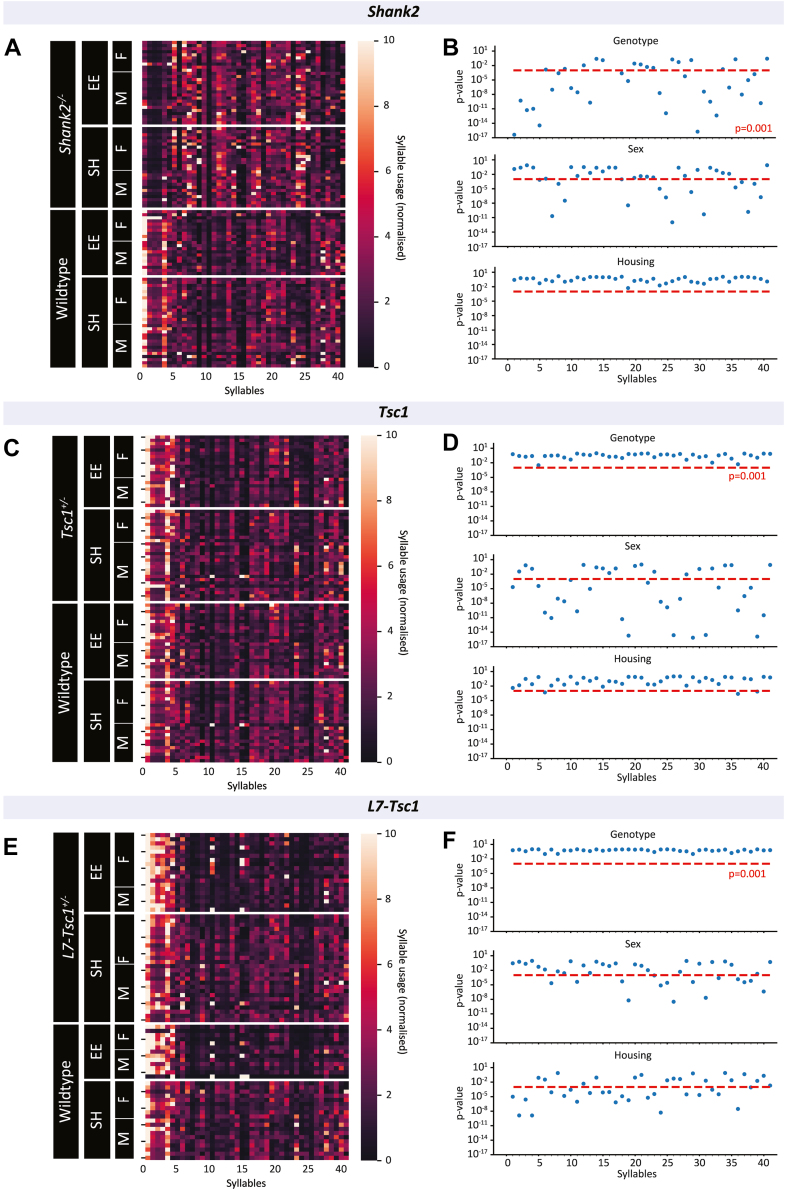
Unsupervised segmentation of behavior of autism models in an open field arena using motion sequencing. **(A, C, E)** Heatmaps of normalized syllable usages per genotype. Each row represents an individual animal, and each column represents a behavioral syllable. **(B, D, F)** Significance of syllables for sex, housing, and genotype differences, with the described feature of each subfigure as the only variable, obtained by 3-way analysis of variance. A *p* value of .001 was calculated based on the number of variables and applied to correct for multiple comparisons using the Šidák correction method. Each dot represents a syllable. *Shank2*, *n* = 103; *Tsc1*, *n* = 98; *L7-Tsc1*, *n* = 77 mice. EE, environmentally enriched; F, female; M, male; SH, standard housing.

A total of 41 behavioral syllables were identified through MoSeq (Figure 2A, C, E). In *Shank2*^*−/−*^ mice, expression of behavioral syllables showed significant alterations in their usage depending on the genotype (Figure 2B, top) and sex (Figure 2B, center). However, no syllables were found to be differentially expressed by *Shank2*^*−/−*^ mice raised in different environmental conditions (Figure 2B, bottom). This was surprising given that the classical elevated plus maze clearly showed significant differences in behavior dependent on environmental conditions. In the *Tsc1*^*+/−*^ mice, MoSeq was able to detect sex-specific expression of behavioral syllables (Figure 2D, center), yet no genotype- or environment-specific syllables were found (Figure 2D, top and bottom). Similarly, *L7-Tsc1*^*flox/+*^ mice showed differential expression of sex-specific syllables but no genotype-specific syllables (Figure 2F, top and bottom). However, behavioral syllables specific to environmental conditions were clearly displayed in *L7-Tsc1*^*flox/+*^ mutant mice (Figure 2F, bottom). Characterization of the transitional structure of behavioral syllables showed increased transition probabilities in *Shank2*^*−/−*^ mice compared with WT littermates (Figure S4A). These results indicate the ability of MoSeq to accurately capture sex differences in all tested models, with additional descriptive power for genotype differences in *Shank2*^*−/−*^ mice and environmental effects in heterozygous *L7-Tsc1*^*flox/+*^ mice.

### Automated Profiling of Group Behaviors in Autism Models Reveals Sex- and Environment-Dependent Motor and Social Phenotypes

**Figure 3:**
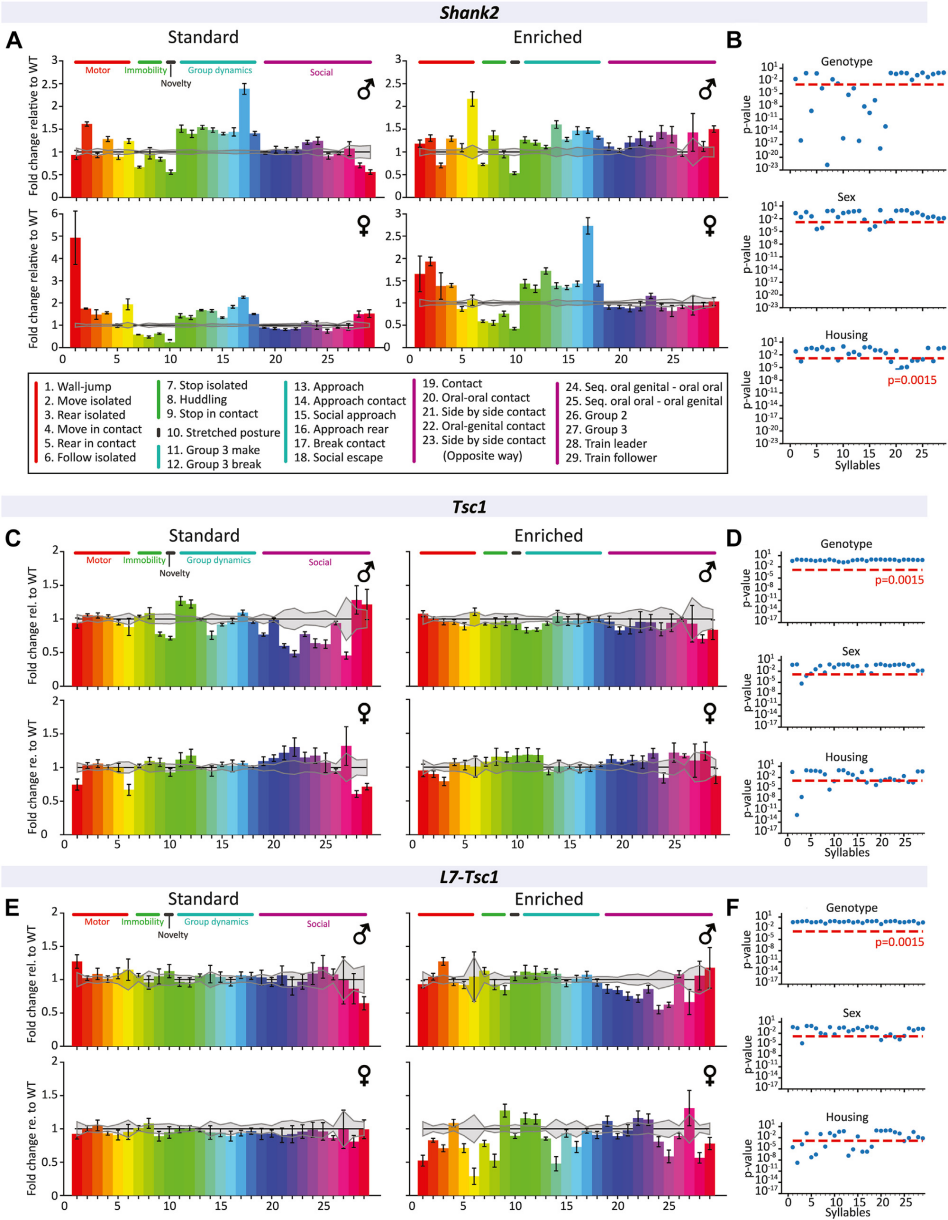
Behavioral analysis of autism mouse models in a home-cage environment. **(A, C, E)** Fold change relative to respective WT results. Gray error lines indicate standard error deviation from WT mean. Data presented as mean with SEM. Each bar represents a behavior. **(B, D, F)** Significance of behaviors for sex, housing, and genotype differences, with the described feature of each subfigure as the only variable, obtained by 3-way analysis of variance. Each dot represents a syllable. A *p* value of .0015 was calculated based on the number of variables and applied to correct for multiple comparisons using the Šidák correction method. *Shank2*, *n* = 91; *Tsc1*, *n* = 88; *L7-Tsc1*, *n* = 87 mice. WT, wild-type.

The LMT (22) allows for automated quantification of group behaviors in a home cage–like environment, whereas previously mentioned behavioral assays only quantify the behavior of single animals. This allows for more naturalistic expression of social behaviors. Similar to the performance in classical behavioral assays, *Shank2*^*−/−*^ mice exhibited increased motor-related behaviors and decreased immobility-related behaviors in the LMT, reflecting the hyperactive phenotype we observed in the open field test (Figure 3A). Similarly, transition probabilities between behaviors in the LMT were larger in *Shank2*^*−/−*^ mice than WT littermates (Figure S4B), further showcasing the hyperactivity phenotype in these animals. Similar to results in the social chamber test, where *Shank2*^*−/−*^ mice exhibited normal levels of social behavior (Figure S3B), expression of most social behaviors in the LMT was found to be intact (Figure 3A). Interestingly however, behaviors related to group dynamics were strongly dysregulated, indicating normal levels of sociability but differential, more dynamic expression of individual social bouts. *Shank2*^*−/−*^ mice showed significant genotype-, sex-, and environment-specific differences in several group and individual behaviors compared with WT littermates (Figure 3B and Figure S5). *Tsc1*^*+/−*^ mice displayed less pronounced phenotypic differences compared with *Shank2*^*−/−*^ mice. Differential expression of social behavior was found between male and female animals raised in standard housing, with female *Tsc1*^*+/−*^ mice generally expressing higher sociability and male *Tsc1*^*+/−*^ mice displaying significant deficits in social behavior (Figure 3C). Furthermore, environmental conditions significantly modulated several motor-related behaviors and social behaviors in *Tsc1*^*+/−*^ mice, whereas sex only showed minor impact on measured behaviors, suggesting that differences in this model could be primarily environment dependent and to a lesser degree sex dependent (Figure 3D and Figure S3). In *L7-Tsc1*^*flox/+*^ mice, no significant difference in behaviors was found for genotype and sex (Figure 3E, F and Figure S3). However, animals raised in enriched environments showed a significantly different behavioral profile compared with standard housed *L7-Tsc1*^*flox/+*^ mutant mice (Figure 3E, F).

### Combining Classical and Multiparametric Measurements Achieves the Highest Classification Accuracy Across All Mouse Models

**Figure 4:**
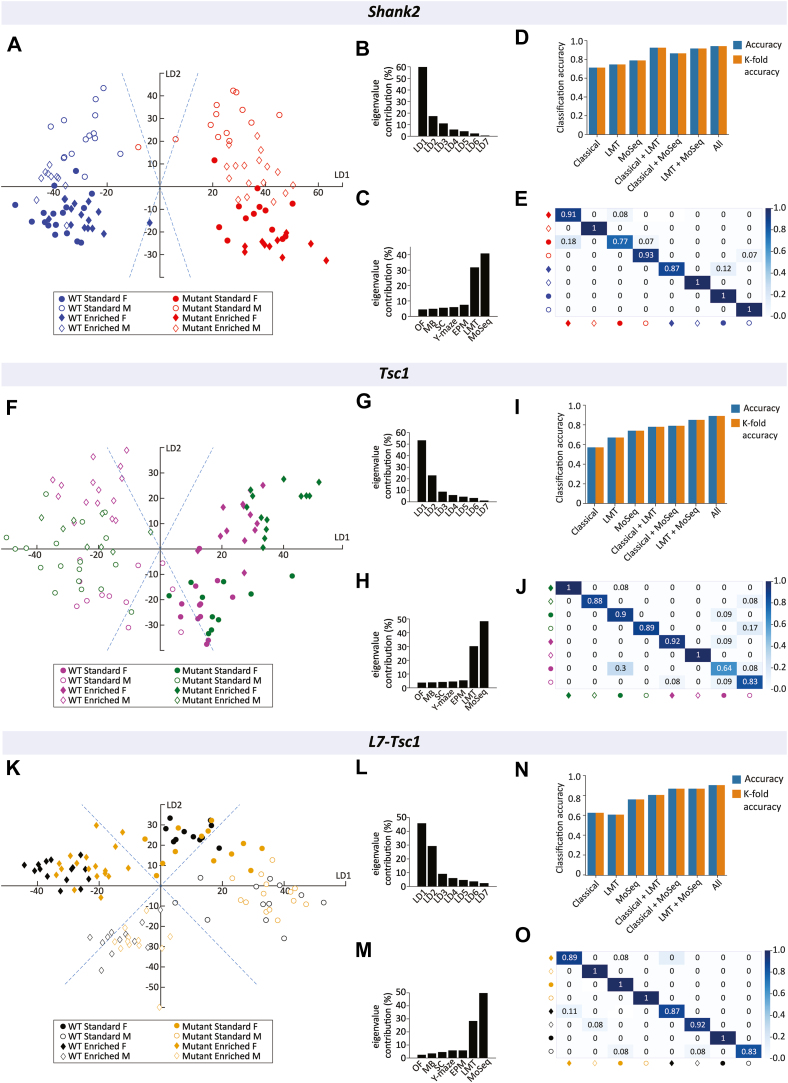
Classification accuracy of classical and multiparametric data with reduced dimensionality. **(A, F, K)** Two-dimensional representation of the first 2 LDs with the highest eigenvalue contribution, indicating the amount of variance captured. The x-axis represents LD1, the LD with the highest eigenvalue contribution, and the y-axis represents LD2, the LD with the second highest eigenvalue contribution. Standard housing conditions are identified by circles, while enriched housing conditions are represented using diamonds. Female and male mice are distinguished by filled and open symbols, respectively. **(B, G, L)** Eigenvalue contribution of all LDs, indicating the captured variance. **(C, H, M)** Eigenvalue contribution of separate behavioral assays. **(D, I, N)** Accuracy and cross-validation for different combinations of behavioral assays used to train the linear support vector machine classifier. The y-axis shows the classification accuracy of the linear classifier and the accuracy result from leave-one-out cross-validation. **(E, J, O)** Confusion matrix of experimental groups made by incorporating behavioral data from classical and multiparametric behavioral assays. The y-axis represents the true mice group the animals belong to, while the x-axis is the group which the classification model classified the mice as. Values in the confusion matrix represent the ratios of group prediction for the true groups. *Shank2*, *n* = 119; *Tsc1*, *n* = 100; *L7-Tsc1*, *n* = 109 mice. EPM, elevated plus maze; F, female; LD, linear discriminant; LMT, Live Mouse Tracker; M, male; MB, marble burying; MoSeq, motion sequencing; OF, open field; SC, social chamber; WT, wild-type.

Classical behavioral assays, LMT, and MoSeq have each shown the ability to quantify distinct aspects of the behavioral phenotypes. To further examine the capabilities of classical and multiparametric assays to capture the modulating effect of environment and sex on the behavior of autism mouse models, we performed principal component analysis–linear discriminant analysis and machine learning classification on data collected across all assays. In total, 20 experimental measures were obtained from classical behavioral testing, information on 41 behavioral syllables was obtained from MoSeq, and 29 measures were obtained from the LMT. Two-dimensional representation of the first 2 linear discriminants with the highest eigenvalue showed a clear separation between *Shank2* experimental groups (Figure 4A). The first linear discriminant best separates *Shank2*^*−/−*^ mice and their WT littermates (Figure 4A), explaining a substantial amount of the variability in the data (60%) (Figure 4B). Quantification of the variance explained per experiment shows that most of the explained variance between classes originates from MoSeq and LMT analyses (MoSeq, 41%; LMT, 32%) (Figure 4C), indicating that these multiparametric tests are highly capable of differentiating between *Shank2* experimental groups. To fully identify the ability of the classical and multiparametric behavioral assays to differentiate sex, environmental conditions, and genotypes, a linear support vector machine classifier was trained for a multitude of combinations of classical and multiparametric assays. Individually, classical and multiparametric assays only reached classification rates between 65% and 75%, whereas combining behavioral assays yielded substantial gains in classification accuracy (96%) (Figure 4D). Combining the various assays showed a clear ability to accurately classify experimental groups in *Shank2*^*−/−*^ mice and their WT littermates (Figure 4E). In *Tsc1*^*+/−*^ and WT mice, visual representation of the first 2 linear discriminants shows clear segmentation between sexes by the first linear discriminant (Figure 4F), with a higher contribution to the total variability in the data of additional linear discriminants than *Shank2* experimental groups (Figure 4G). LMT and MoSeq captured large amounts of the variability in experimental groups (MoSeq, 48%; LMT, 30%) (Figure 4H). Linear support vector machine classifiers showed clear gains in classification accuracy by combining classical and multiparametric behavioral assays (92%) (Figure 4I), in line with the more evenly distributed contribution of additional linear discriminants. This resulted in an accurate classification of most *Tsc1*^*+/−*^ and WT experimental groups (Figure 4J). In *L7-Tsc1*^*flox/+*^ mice and their WT littermates, the first 2 linear discriminants show strong separation between sexes and the different environmental conditions (Figure 4K, L). Similar to the previous models, most of the variance in the experimental groups, which in this case is mostly related to variability due to environmental conditions, can be attributed to MoSeq and LMT analyses (MoSeq, 50%; LMT, 28%) (Figure 4M). A combination of classical and multiparametric assays showed a high level of classification accuracy (95%) (Figure 4N, O).

**Figure 5:**
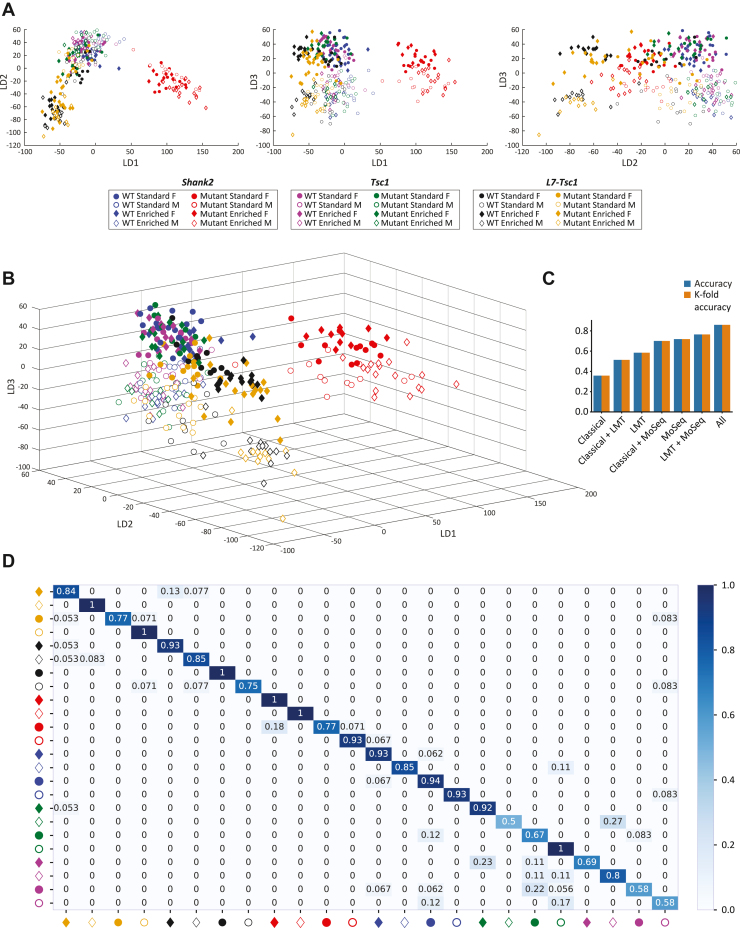
Classification accuracy for combined data of all genotypes. **(A)** Two-dimensional representation of the first 3 LDs with the highest eigenvalue contribution, indicating the amount of variance captured. **(B)** Three-dimensional representation of the first 3 LDs with the highest eigenvalue contribution. The x-axis represents LD1, the LD with the highest eigenvalue contribution; the y-axis represents LD2, the LD with the second highest eigenvalue contribution; and the z-axis represents LD3, the LD with the third highest eigenvalue contribution. **(C)** Accuracy and cross-validation for different combinations of behavioral assays used to train the linear support vector machine classifier. The y-axis shows the classification accuracy of the linear classifier and the accuracy result from leave-one-out cross-validation. **(D)** Confusion matrix showing all of the tested autism models and subgroups made by incorporating behavioral data from classical and multiparametric behavioral assays. The y-axis represents the true mice group the animals belong to, while the x-axis is the group which the classification model classified the mice as. Values in the confusion matrix represent the ratios of group prediction for the true groups. *Shank2*, *n* = 119; *Tsc1*, *n* = 100; *L7-Tsc1*, *n* = 109 mice. F, female; LD, linear discriminant; LMT, Live Mouse Tracker; M, male; MoSeq, motion sequencing; WT, wild-type.

Additionally, when behavioral data from all genotypes were combined (Figure 5A, B), classification accuracy showed a linear increase in performance with combined data from classical and multiparametric assays (Figure 5C). Per-group classification showed especially high predictive validity between the genotypes, with higher confusion rates mostly restricted to comparisons within genotypes (Figure 5D). Together, these results indicate strong gains in classification accuracy of experimental groups by dimensionality reduction and a combination of both classical and multiparametric behavioral assays.

## Discussion

Autism is a complex neurodevelopmental disorder that exhibits high genetic and phenotypic variability (7,12). Here, we integrated classical and multiparametric assays to explore the spectrum of phenotypic variability in 3 commonly used autism mouse models, taking sex and environmental modifiers into account.

We observed that classical behavioral assays were capable of accurately capturing several key aspects of previously reported behavioral deficits in *Shank2*^*−/−*^ mice (41,49,58). In our battery, *Shank2*-deficient mice exhibited anxiety-related behaviors, hyperactivity, and learning deficits. Growing up in enriched housing conditions significantly modulated the exploratory behavior of *Shank2*^*−/−*^ mice in the elevated plus maze, emphasizing the impact of environmental conditions on behavioral phenotypes. Classical behavioral assays showed a lack of clear impairments in the *Tsc1*^*+/−*^ and heterozygous *L7-Tsc1*^*flox/+*^ mice, which is in contrast with previous findings that showed social impairments in these lines (45,59). We attribute this discrepancy to 3 possible factors. First, our mouse lines were fully backcrossed into the C57BL/6J background; published studies with *L7-Tsc1*^*flox/+*^ used mice on mixed backgrounds (45). Because different background strains are known to have considerable phenotypic variability (60), this could explain some of the observed differences. Second, environmental factors have been shown to affect behavioral outcomes (61); therefore, differences in experimental conditions such as prior exposure to other tests or differences in animal housing and handling may have contributed to the poor reproducibility of deficits in *Tsc1*^*+/−*^ and *L7-Tsc1*^*flox/+*^ mice. Third, it is possible that for mice with relatively mild and variable phenotype such as the *Tsc1*^*+/−*^ and *L7-Tsc1*^*flox/+*^, failure to detect a significant difference between the genotypes in the classical assays can simply reflect the genetic heterogeneity within the strain itself. Between 30% and 60% of people who harbor loss-of-function mutations in the *TSC1* and *TSC2* genes receive an autism diagnosis (62), with mutations in the *TSC2* gene showing a higher risk (63). Therefore, it is plausible that in those cases, we need more refined tests to capture the diversity of behavioral phenotypes.

Next to classical behavioral tests, we used 2 techniques—LMT (22) and MoSeq (23)—to assess their ability to discern subtle behavioral impairments in tested mouse models. While the LMT enables identification of predetermined single and group behaviors for multiple mice using supervised learning, MoSeq dynamically builds representations of movement for individual mice using unsupervised machine learning. We have chosen not to assign labels to the individual syllables, in line with previous publications using MoSeq (23,56), and rather to keep them as an abstract representation of mouse behavior.

The automated profiling of home cage–like behaviors using the LMT unveiled sex- and environment-dependent motor and social phenotypes. *Shank2*^*−/−*^ mice exhibited a hyperactive phenotype consistent with classical assays and previous studies (49). *Shank2*^*−/−*^ mice also exhibited differential expressions of behaviors related to social group dynamics consistent with previous reports (41,49). Similarly, *Tsc1*^*+/−*^ mice showed nuanced sex-dependent social behaviors and environment-dependent motor behaviors. *L7-Tsc1*^*flox/+*^ mice exhibited distinct behavioral profiles under enriched housing conditions, highlighting the interplay between genetic factors and environmental influences in shaping behavioral outcomes.

MoSeq, which previously had been used for pharmacological studies (56) and research on a cerebellar-specific autism mouse model (25), was able to recognize genotype- and sex-specific behavioral syllables in *Shank2*^*−/−*^ mice in our study. However, none of the 41 most used syllables were significantly affected by environmental conditions in these mice. It is possible that large genotype and sex differences in *Shank2*^*−/−*^ mice are so dominant that they simply occlude other characteristics. The presence of sex-specific expression of behavioral syllables in all of the models further reinforces the importance of considering sex differences in autism research (31).

The integration of classical behavioral assays, MoSeq, and LMT allowed for a comprehensive assessment of behavioral phenotypes in all of the tested models. Machine learning classification further demonstrated the high predictive accuracy of a multiparametric approach in quantifying the impact of genotype, sex, and environmental conditions on behavioral phenotypes. Although it is hard to ascribe translational functionality to single behavioral outcomes, the integration of multidimensional behavioral analyses of mouse models of human disorders has translational potential in their ability at quantifying the variability inherent to many disorders (64). This is particularly important given the ongoing discussion on using individualized assessments for therapeutic and prognostic purposes in humans (65). Technology-aided, personalized assessments (e.g., data from smartphones and wearables) are being rapidly integrated into clinical practice, used for both initial diagnosis and tracking progress during targeted therapeutic intervention (66).

Notably, in our analysis, we have focused on environmental enrichment and sex differences, leaving out many other developmental aspects that were shown to contribute to behavioral variability in autism, such as stress and maternal infection (67,68) or maternal separation (69). We chose to study environmental enrichment because it has been used often in the past as a form of rescue of the behavioral deficits in autism mouse models (27, 28, 29,70) and is becoming the standard form of husbandry in some animal facilities, in line with the 3 Rs (replacement, reduction, and refinement) principles (71,72). Finally, in our current work, we have focused on adult phenotypes, but to fully understand the behavioral variability of autism phenotypes, future research needs to include longitudinal behavioral assessment, similar to longitudinal human cohorts (73). It remains to be explored how multiparametric tests can serve this purpose.

We recognize that the preferred combination of assays may vary depending on the severity of the behavioral impairments and the existence of environmental modifiers. For the quantification of severe deficits, it could be sufficient to use specific, single-trait assays. However, for milder, complex phenotypes, the combination of LMT and MoSeq offers an in-depth description of the behavioral repertoire. Here, we used homozygous *Shank2*^*−/−*^ mice and heterozygous global and Purkinje cell–specific *Tsc1*^*+/−*^ mice, showing the efficacy of these methods on a range of phenotypic severities. It will be of interest for future studies to investigate gene-dosage effects using homozygous and heterozygous mutant mice of the same line in a similar experimental design combining LMT and MoSeq analysis.

### Conclusions

Our multiparametric approach offers insights into the interplay between genetics, sex, and environment in autism mouse models. These findings contribute to the evolving understanding of the heterogeneity of autism and underscore the importance of comprehensive behavioral assessments in preclinical research.

## Supplementary Material

Key Resources Table
