# Supplementary material for: Multiparametric Assays Capture Sex- and Environment-Dependent Modifiers of Behavioral Phenotypes in Autism Mouse Models
Source: Biol Psychiatry Glob Open Sci. 2024 Jul 20;4(6):100366. doi: 10.1016/j.bpsgos.2024.100366 (PMC11387692; doi:10.1016/j.bpsgos.2024.100366)
Supplement: Supplementary Material [file mmc2.pdf]

SUPPLEMENTARY INFORMATION

Multiparametric Assays Capture Sex- and Environment-Dependent Modifiers of Behavioral Phenotypes in Autism Mouse Models

Wahl *et al.*

Supplementary results

Supplementary figure 1

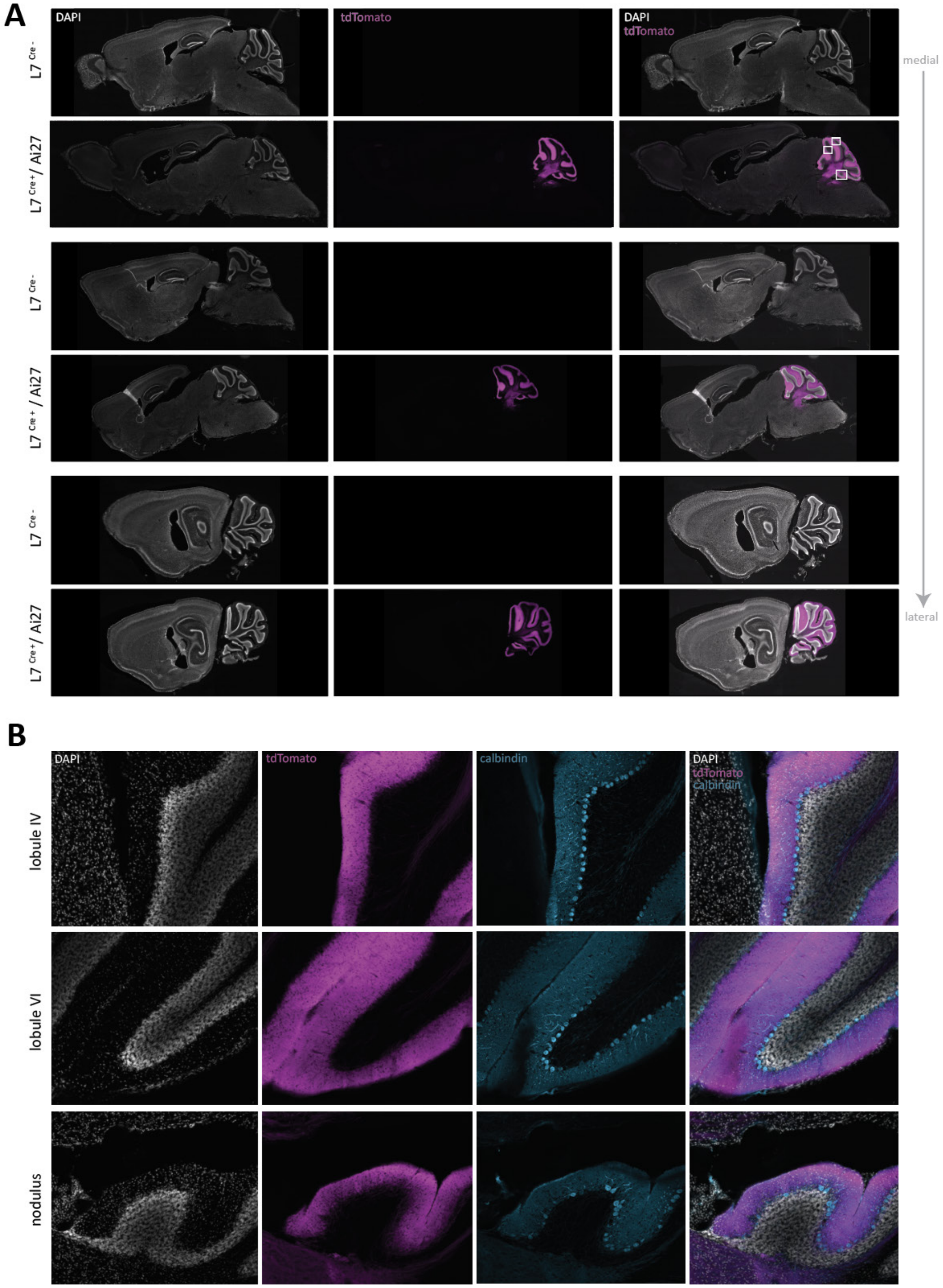

Supplementary figure 1. Cre recombinase expression in the *L7Cre*$^+$ mouse line is restricted to Purkinje cells. A) Sagittal sections of the entire brains of adult *L7Cre*$^+$*/Ai27* and *L7Cre*$^-$ mice. Note the lack of tdTomato expression anywhere outside of the cerebellum in the *L7Cre*$^+$*/Ai27* mouse. White - DAPI; magenta - tdTomato. **B**) Magnified images of the cerebellum of the *L7Cre*$^+$*/Ai27* mouse, indicated in **A** by the squares (second row, third column image). Overlay of the calbindin (Purkinje cell marker) and the tdTomato signals shows that the *Cre* expression in *L7Cre*$^+$*/Ai27* mouse is restricted to the molecular layer across the entire cerebellar cortex. White - DAPI; magenta - tdTomato; light blue - calbindin.

Supplementary figure 2

				MoSeq	LMT	Machine Learning*	Social chamber	Elevated plus maze	Open field	Y-maze	Marble burying
Shank2	**Wt**	**Std**	**F**	15	12	17	18	18	18	15	13
			**M**	14	11	15	11	14	14	10	11
		**EE**	**F**	10	10	15	13	13	13	9	9
			**M**	11	11	13	13	13	9	10	7
	**Mutant**	**Std**	**F**	14	11	13	14	15	15	5	12
			**M**	12	11	14	13	13	13	8	9
		**EE**	**F**	10	11	11	11	11	11	9	7
			**M**	17	14	21	17	17	11	10	12
Tsc1	**Wt**	**Std**	**F**	13	12	12	12	12	12	10	12
			**M**	12	13	12	13	13	13	12	13
		**EE**	**F**	12	10	13	13	11	13	9	12
			**M**	11	12	15	16	16	16	13	16
	**Mutant**	**Std**	**F**	10	10	9	10	11	11	5	11
			**M**	18	11	18	17	17	17	15	15
		**EE**	**F**	13	12	13	15	14	14	12	14
			**M**	9	8	8	12	12	12	11	12
**L7-*Tsc1***	**Wt**	**Std**	**F**	9	10	11	11	11	11	9	9
			**M**	11	7	12	12	12	12	11	11
		**EE**	**F**	6	11	15	16	16	14	12	13
			**M**	6	11	13	10	10	10	9	9
	**Mutant**	**Std**	**F**	12	12	13	13	13	13	11	12
			**M**	14	13	14	12	13	12	12	10
		**EE**	**F**	13	13	19	19	19	15	17	14
			**M**	6	10	12	11	11	11	10	9

Supplementary figure 2. Number of animals used in behavioral testing. The number of animals used per test for classical and multi-parametric behavioral assays. *Data used in dimensionality reduction and machine learning analysis, consisting of data from MoSeq, LMT and single-trait behaviors.

Supplementary figure 3

A

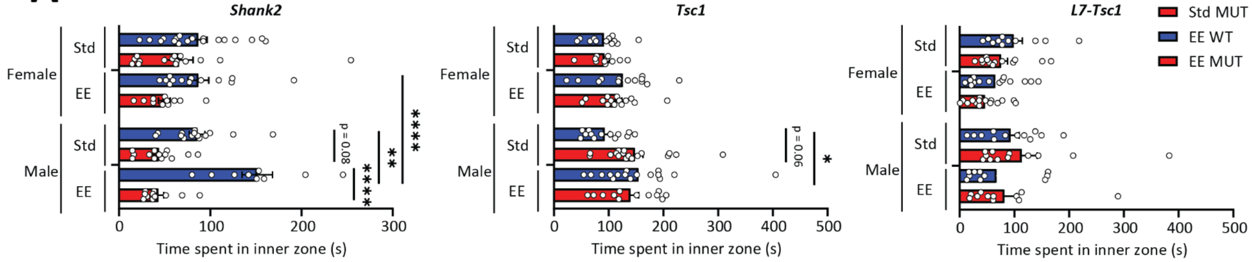

	Shank2			*Tsc1*			*L7-Tsc1*		
Source of Variation	% of total variation	P value	P value summary	% of total variation	P value	P value summary	% of total variation	P value	P value summary
Sex	0.8133	0.2639	ns	5.968	0.0078	**	2.2028	0.1548	ns
Housing conditions	1.602	0.1181	ns	5.872	0.0083	**	6.011	0.0154	*
Genotype	28.57	<0.0001	****	0.4200	0.4739	ns	0.02195	0.8817	ns
Sex x Housing conditions	4.317	0.0111	*	0.003942	0.9445	ns	0.01371	0.9064	ns
Sex x Genotype	5.514	0.0043	**	1.326	0.2036	ns	2.432	0.1196	ns
Housing conditions x Genotype	4.489	0.0097	**	3.268	0.0472	*	0.0007937	0.9774	ns

B

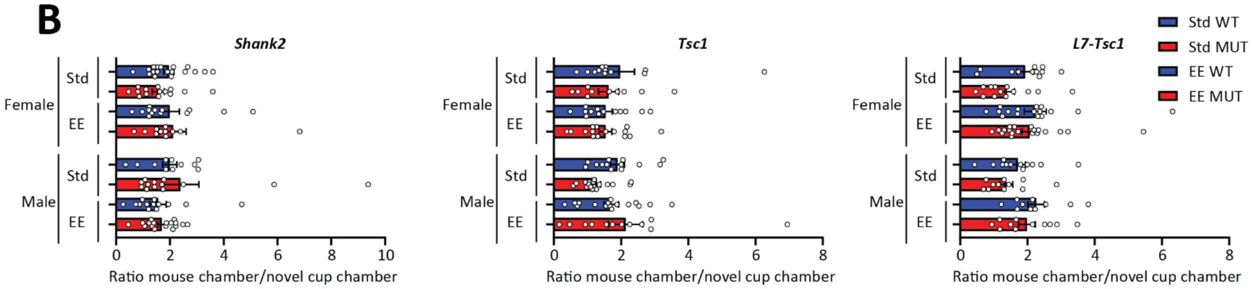

	Shank2			*Tsc1*			*L7-Tsc1*		
Source of Variation	% of total variation	P value	P value summary	% of total variation	P value	P value summary	% of total variation	P value	P value summary
Sex	9.014e-005	0.9922	ns	0.1674	0.6725	ns	0.1448	0.6941	ns
Housing conditions	0.2905	0.5790	ns	0.04022	0.8358	ns	7.716	0.0049	**
Genotype	0.06887	0.7869	ns	0.4345	0.4961	ns	3.004	0.0756	ns
Sex x Housing conditions	2.751	0.0898	ns	2.153	0.1315	ns	0.05226	0.8132	ns
Sex x Genotype	0.6164	0.4194	ns	0.03544	0.8457	ns	0.01603	0.8959	ns
Housing conditions x Genotype	0.05697	0.8058	ns	2.931	0.0791	ns	0.3338	0.5506	ns

C

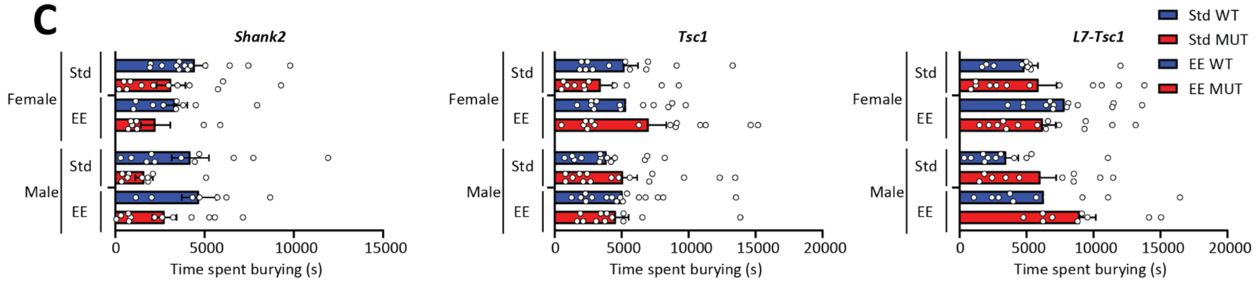

	Shank2			*Tsc1*			*L7-Tsc1*		
Source of Variation	% of total variation	P value	P value summary	% of total variation	P value	P value summary	% of total variation	P value	P value summary
Sex	0.002176	0.9659	ns	0.7194	0.3854	ns	1.599e-0.005	0.9969	ns
Housing conditions	0.03058	0.8728	ns	2.435	0.1119	ns	8.385	0.0064	**
Genotype	10.96	0.0033	**	0.04823	0.8219	ns	2.246	0.1509	ns
Sex x Housing conditions	2.955	0.1186	ns	1.147	0.2736	ns	0.5982	0.4563	ns
Sex x Genotype	1.008	0.3593	ns	0.09073	0.7575	ns	3.409	0.0778	ns
Housing conditions x Genotype	0.1863	0.6928	ns	0.3787	0.5285	ns	0.6532	0.4364	ns

Supplementary figure 3. Behavioral phenotyping of autism mouse models using classical behavioral assays. A) Time spent in the inner zone of an open field test in *Shank2* mice (left), Tsc1 mice (center) and *L7-Tsc1* mice (right). Data presented as mean with SEM (three-way ANOVA with Holm-Sidak's multiple comparisons test). **B)** Ratio of the time spent in the chamber containing a novel mouse divided by the time spent in the chamber with a novel object in the social chamber test, in *Shank2* mice (left), *Tsc1* mice (center) and *L7-Tsc1* mice (right). Data presented as mean with SEM (three-way ANOVA with Holm-Sidak's multiple comparisons test). **C)** Time spent burying during the marble burying assay in *Shank2* mice (left), *Tsc1* mice (center) and *L7-Tsc1* mice (right). Data presented as mean with SEM (three-way ANOVA with Holm-Sidak's multiple comparisons test). * $p \leq 0.05$, ** $p \leq 0.01$, *** $p \leq 0.001$

Supplementary figure 4

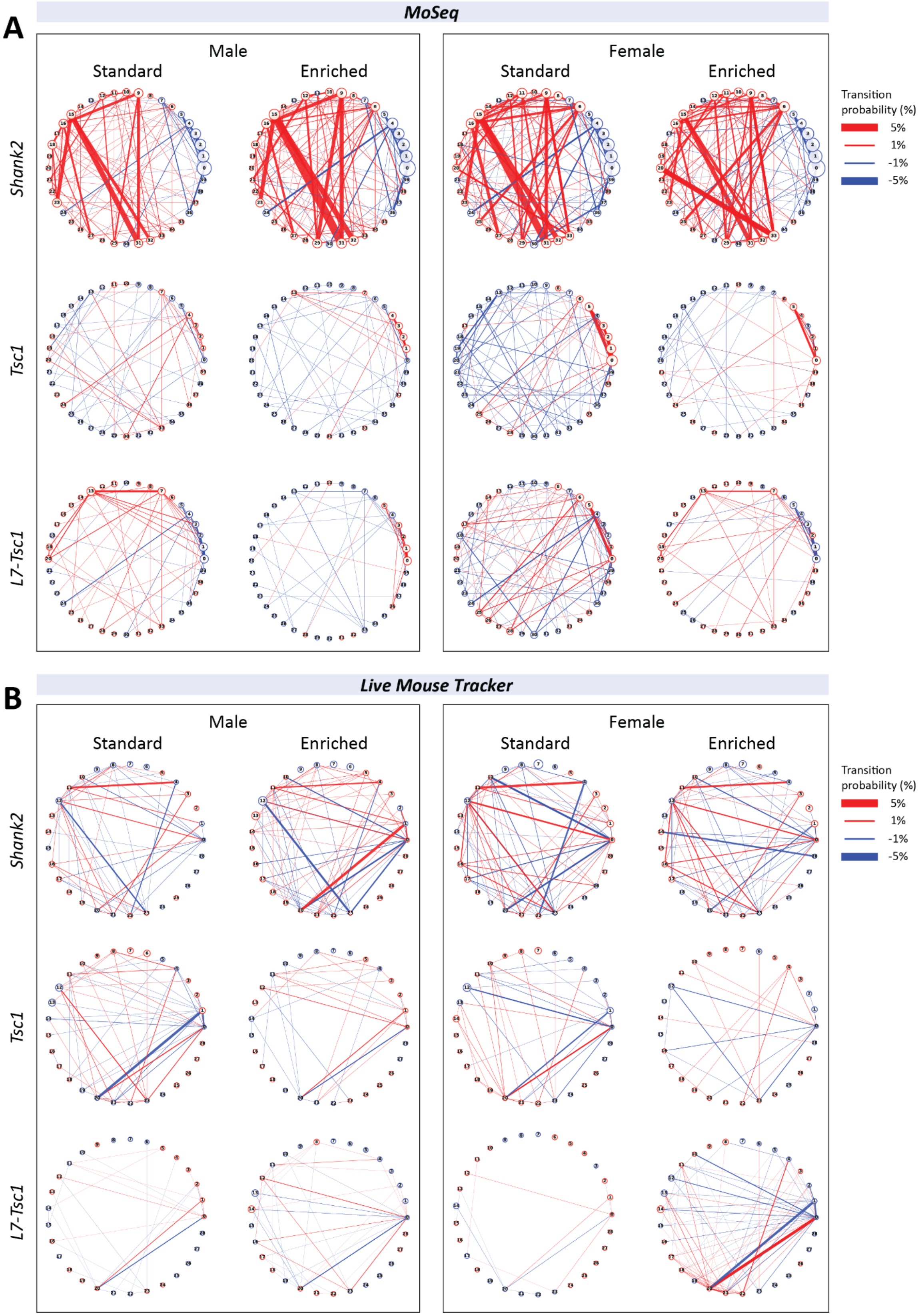

Supplementary figure 4. Probabilities of transition between behaviors in the MoSeq and LMT. Red lines indicate increased transition probabilities in mutant animals, whereas blue lines indicate decreased transition probabilities. Line thickness indicates the probability size, depicting transitions greater than 0.5% and smaller than -0.5% in size respectively. *Shank2*, n = 91; *Tsc1*, n = 88; *L7-Tsc1*, n = 87 mice.

Supplementary figure 5

	Shank2			*Tsc1*			*L7-Tsc1*		
	Genotype	Sex	housing	Genotype	Sex	housing	Genotype	Sex	housing
WallJump	0.031	0.202	0.060	0.401	0.565	0.312	0.450	0.476	<0.001
Move isolated	<0.001	0.025	<0.001	0.953	0.750	<0.001	0.583	0.241	<0.001
Rear isolated	0.732	0.368	0.245	0.771	<0.001	<0.001	0.753	<0.001	<0.001
Move in contact	<0.001	0.036	0.403	0.730	0.001	0.885	0.977	0.922	0.150
Rear in contact	0.638	<0.001	0.200	0.597	0.002	0.666	0.422	0.955	<0.001
FollowZone Isolated	<0.001	<0.001	0.598	0.405	0.035	0.644	0.469	0.210	<0.001
Stop isolated	<0.001	0.661	0.139	0.611	0.332	0.434	0.702	0.011	0.098
Huddling	0.025	0.784	0.238	0.431	0.005	0.073	0.193	0.005	<0.001
Stop in contact	0.003	0.024	0.001	0.985	0.735	0.000	0.764	0.456	0.172
Stretched attend posture	<0.001	0.212	0.897	0.539	0.121	0.001	0.919	0.005	0.037
Group 3 make	<0.001	0.372	0.015	0.223	0.688	0.888	0.513	0.264	0.402
Group 3 break	<0.001	0.561	0.043	0.163	0.696	0.934	0.553	0.365	0.565
Approach	<0.001	0.713	0.010	0.217	0.758	0.147	0.872	0.034	<0.001
Approach contact	<0.001	0.006	0.567	0.563	0.201	0.055	0.365	0.076	<0.001
Social approach	<0.001	<0.001	0.609	0.747	0.277	0.004	0.646	0.245	0.022
Approach rear	<0.001	<0.001	0.117	0.907	0.007	0.275	0.275	0.074	<0.001
Break contact	<0.001	0.996	0.107	0.381	0.363	<0.001	0.898	0.525	<0.001
Social escape	<0.001	0.002	0.024	0.949	0.003	0.979	0.724	0.456	<0.001
Contact	0.822	0.004	<0.001	0.870	0.602	<0.001	0.969	0.201	0.892
Oral-oral Contact	0.475	0.760	0.085	0.991	0.867	0.001	0.138	0.000	0.743
Side by side Contact	0.910	0.905	<0.001	0.767	0.435	0.003	0.650	0.002	0.843
Side by side Contact, opposite way	0.728	0.900	<0.001	0.912	0.346	0.006	0.769	0.005	0.973
Oral-genital Contact	0.019	0.178	<0.001	0.819	0.548	0.002	0.994	0.001	0.308
seq oral geni - oral oral	0.240	0.315	<0.001	0.427	0.648	0.001	0.440	<0.001	0.138
seq oral oral - oral genital	0.927	0.075	0.001	0.854	0.852	0.025	0.442	0.002	0.001
Group2	0.088	0.019	0.393	0.830	0.288	0.001	0.656	0.217	0.008
Group3	0.434	0.032	<0.001	0.787	0.693	<0.001	0.874	0.062	0.351
TrainLeader	0.856	0.011	0.285	0.685	0.015	0.444	0.218	0.164	0.014
TrainFollower	0.949	0.014	0.419	0.687	0.016	0.453	0.385	0.161	0.007

Supplementary figure 5. Significance of behaviors in the LMT for sex, housing and genotype differences. Obtained by 3-way ANOVA. A p value of 0.0015 was calculated based on the number of variables and applied to correct for multiple comparisons. Significant cells are highlighted in light red. n = 266 mice.

Supplementary Materials and Methods

Genotyping of the transgenic lines

Transgenic mouse model *CrB6.Cg-Tg(Pcp2-cre)3555Jdhu/J::Tsc1*$^{flox/+}$ (*L7-Tsc1*$^{flox/+}$) was achieved by crossing *Tsc1*$^{flox/flox}$ (Jackson Laboratory RRID:IMSR_JAX:005680) females with *B6.Cg-Tg(Pcp2-cre)3555Jdhu/J* (MGI:3053828, referred to as *L7Cre*) males. New litters were genotyped using toe tissue between 4-7 days old and re-genotyped after finishing all experiments. *Tsc1*$^{flox/flox}$ PCR genotyping was conducted by amplification of 30 cycle on a 58 degrees protocol using the following three primers: (oIMR4008) forward pimer 5' GTCACGACCGTAGGAGAAGC 3', (oIMR4009) reverse primer 5' GAATCAACCCCACAGAGCAT 3' and to identify possible global deletions, only present after crossing with a Cre-line, we used the third primer 5' AGGAGGCCTCTTCTGCTACC 3'. The PCR products gave band sizes of 190 bp (wildtype), 230 bp (mutant) and 400 bp (deletion). The *L7Cre* strain was genotyped using the same protocol using the four primers: (oIMR1084) forward primer 5' GCG GTC TGG CAG TAA AAA CTA TC 3', (oIMR1085) reverse primer 5' GTG AAA CAG CAT TGC TGT CAC TT 3', (oIMR7338) internal positive control forward primer 5' CTA GGC CAC AGA ATT GAA AGA TCT 3' and the (oIMR7339) internal positive control reverse primer 5' GTA GGT GGA AAT TCT AGC ATC ATC C 3'. The band sizes were 100bp (Cre transgene) and 324 bp (internal positive control). Behavioral experiments were performed on *Cre* positive heterozygous for *Tsc1* transgenic mice of both sexes.

The 5' AGGAGGCCTCTTCTGCTACC 3' primer was used for genotyping of the *Tsc1*$^{tm1.1Djk}$ (MGI:2183900; *Tsc1*$^{+/-}$) strain. For this line heterozygotic *Tsc1*$^{+/-}$ male mice were crossed with *C57BL/6J* females.

The *B6N.129S4-Shank2*^{tm1Mgle}*/CsbdJ* (*Shank2*$^{-/-}$) strain. For this strain, the following three primers were used in a 30 cycle, 58 degrees PCR reaction: (1) wildtype forward primer 5' GCT AGC ATG ACG TGT GTT GTG 3', (2) mutant forward primers 5' CCG ACT GCA TCT GCG TGT TC 3' and (3) reverse primer 5' ACC TGT GTG TGA TTT CTG AC 3' giving the PCR products of ± 525 bp (wildtype) and ± 591 bp (mutant). Heterozygous F1 *Shank2*$^{+/-}$ females were bred with heterozygous F1 males to achieve homozygous *Shank2*$^{-/-}$deletion.

All mouse lines were fully backrossed (>10 generations) into the *C57BL/6J* background. At the end of the experiments when animals were euthanized ear tissue was collected and all mice were re-genotyped using the same primers as described above.

Testing CrB6.Cg-Tg(Pcp2-cre)3555Jdhu/J (*L7Cre*) *line expression specificity*

To test the specificity of *Cre* expression in the *L7Cre* line, we crossed the B6.Cg-Gt(ROSA)26Sor$^{tm27.1(CAG-COP4*H134R/tdTomato)Hze/}$J (referred to as *Ai27D*; MGI: 4834650) reporter line with the Purkinje cell specific *Cre* expressing *B6.Cg-Tg(Pcp2-cre)3555Jdhu/J* mice (referred to as *L7Cre*), which we used to create the *L7-Tsc1*$^{flox/+}$ mice. Both strains were kept on a *C57BL/6J* background. We used *L7Cre*$^{+}$ (*Cre* positive; n=1, 130 days old) and *L7Cre*$^{-}$ (*Cre* negative; n=1, 193 days old) mice for histological analysis.

Mice were anesthetized with 0.2 ml pentobarbital (60 mg/ml) and perfused with 0.9% NaCl followed by 4% paraformaldehyde (PFA). Brains were dissected from the skull and stored in 4% PFA at room temperature (rT) for 1.5 hours. They were next changed to a 10% sucrose solution and left overnight at 4°C. Brains were embedded in 12% gelatin and 10% sucrose and left in a solution with 30% sucrose and 4% PFA in PBS at rT for four hours. Next, they were transferred

to a 30% sucrose solution in 0.1 PB and kept at 4°C. Whole brains were sliced at 50 µm with a microtome and slices were kept at 0.1M PB.

Sections were incubated in a blocking solution (10% NHS, 0.5% Triton in PBS) for an hour at rT. After rinsing, sections were incubated for 48 hours at 4°C on a shaker in primary antibody solution with 2% NHS (1:10.000 Calbindin-D 28K$^+$ mouse, C9848, Sigma; 1:2000 anti-NeuN rabbit polyclonal, 4047972, Merck). After rinsing, sections were incubated for 2 hours at rT on a shaker with secondary antibody (1:400 Donkey anti-mouse A488, 715-545-150, Jackson; 1:400 Donkey anti-rabbit Cy5, 711-165-152, Jackson). Sections were counterstained with DAPI. Finally, sections were rinsed in 0.1M PB, placed on coverslips using a solution of 0.1M PB and Chromium(III) potassium sulfate, and mounted on slide glasses with Mowiol®.

Sections were imaged with a Zeiss AxioImager 2 (Carl Zeiss, Jena, Germany) at 10x. Tile scans were taken from whole brain sagittal slices (**Supplementary fig. 1A**). Additional images were taken with the Apotome at 10x and 20x (**Supplementary fig. 1B**).

Additional experimental information on mice used in the experiments

Different sizes of experimental groups are due to the fact that we experienced a prolonged Covid-19 related breeding stop in 2020, which affected the experiments all the way to 2021. Unfortunately, we also experienced a mechanical failure of one of our storage drives, before it was backed up. It was not possible to add more animals to individual tests because of the standard housing and environmental enrichment weaning schedule, which resulted in the experimental time of 10 weeks per cohort. The dimensionality reduction analysis was therefore only used on animals that underwent all tests.

The choice of housing 3 mice per cage was motivated by the size of the standard cages; a larger group could lead to overcrowding and possible fighting among males. Therefore a decision

was made to house 3 mice per cage for all conditions. All experimental mice were always housed in one of the two configurations - 1 mutant mouse with 2 control mice, or 2 mutant mice with one control mouse.

Experimenters were blinded to the genotype of the animals during testing. Genotyping was done prior to the separation of mice into the SH and EE housing, by a lab technician not involved in this study (see acknowledgments). All animals were also re-genotyped after the conclusion of the experiments

Single-trait behavioral assays

All experiments except for the water Y-maze were performed in a behavioral box as described in (1). Single-trait behavioral assays were recorded with a fixed camera (acA 1300-600 gm, Basler AG) positioned above the arena, operated with Bonsai (2) with a frame rate of 25 frames per second (fps). Positional and locomotor analyses were done using the open-source software OptiMouse (3). Animals were habituated to the testing room for 1 hour before experiments. Experimental arenas were cleaned with 70% ethanol between testing of different animals. Data from single-trait behavioral assays was normalized to create the radial plots.

1. *Social chamber test*

A three-chamber social preference test was performed in a 63 × 42.5 × 21 cm transparent acrylic arena divided into three separate chambers, with doors between them. First, the test animal was placed in the central chamber. Next, the doors were opened and the animal could explore all chambers. Lastly, the experimental mouse was guided back into the middle chamber and two novel wire cups were placed in opposite chambers, with one containing a novel mouse. The

experimental animal was left to explore for 10 minutes in each condition. Data was analyzed using Optimouse (3). Variables used in dimensionality reduction analysis were the fraction of time the animal spent in close vicinity of the novel mouse, the fraction of time the animal spent in the chamber with the social partner, the ratio between the time spent in close vicinity with the novel mouse divided by the time spent in the chamber with the novel cup, and the ratio between the time spent in the chamber with the novel mouse divided by the time spent in the chamber with the novel cup.

2. *Elevated plus maze*

The maze consisted of two open and closed arms, each with 29.5 × 8.5 cm white-opaque acrylic base, which were raised off the ground by cylindrical poles 30 cm in height. The closed arms were surrounded by black 20 cm high walls. Mice were placed in the center of a maze and left to explore for 10 minutes. Data was analyzed using Optimouse. Variables used in dimensionality reduction analysis were the time spent in the open arms, the total number of transitions and the distance moved.

3. *Open field test*

Animals were placed in a 50 x 50 x 35 cm white-opaque acrylic arena for 30 minutes. Data was analyzed using Optimouse. Variables used in dimensionality reduction analysis were the time spent in the inner zone, the number of transitions into the inner zone and the distance moved.

4. Marble burying test

The marble burying test was performed as described previously (4). Burying behavior during the test was quantified using JAABA (4). Variables used in dimensionality reduction analysis were the number of burying bouts, the average bout duration and the total burying duration.

5. Y-maze test

A water Y-maze paradigm was used to examine cognitive flexibility (5,6). A Y-shaped opaque polycarbonate apparatus (symmetrical arms: 32 cm length × 9 cm width × 20 cm height), was filled with white, non-toxic, water-based paint (Basic color, 21 white 30081, Creall). On day 0 animals underwent 3 60 s habituation trials. During the acquisition days (Days 1-2), a platform was placed at the end of one of the arms for 4 sessions of 5 consecutive 60 s trials. This location was kept the same on the test day (Day 3; 1 session of 5 trials). Mice were required to have 80% success rate on the test day to progress to the reversal day, with the location of the platform switched. During the reversal (Days 4-5), mice were exposed to 4 sessions of 5 consecutive 60 s trials followed by a 5th "forced" session where a door was placed in the initially learned arm of the maze. Mice were kept in a clean cage under the heating lamp to dry in between the sessions. Videos were recorded with a PsEye camera (Sony) at 30 fps. Data was scored by experimenters. Variables used in dimensionality reduction analysis were the area under the curve (AUC) of the acquisition phase, the AUC of the reversal phase, the number of correct choices, and the number of times the plateau was not reached during a session.

Normalization

Data from single-trait behavioral assays was normalized to create radial plots showing an overview of the behavioral phenotype per autism mouse model. Measures used were: sociability, ratio of time spent near novel object divided by the time spent near novel object in the social chamber test; exploratory behavior, ratio of time spent in open arms divided by time spent in closed arms of the elevated plus maze; anxiety, time spent in inner zone of an open field test; hyperactivity, distance moved in an open field test; learning, area under the curve of the percentage of correct trials during the acquisition phase of the Y-maze; reversal learning, area under the curve of the percentage of correct trials during the reversal phase of the Y-maze; repetitive behavior, total time spent burying during the marble burying test. The data was normalized per test, using $x_{normalized} = (x - x_{minimum}) / (x_{maximum} - x_{minimum})$.

To normalize the LMT data for each mutant group, we divided the time spent in each LMT behavior for the mutant mice with the mean time spent in each LMT behavior for WT mice with the same sex and housing conditions. This created a value of the fold change in behavior for the mutant mice relative to the WT mice.

Dimensionality reduction & machine learning classification

The measurements from the single-trait behavioral assays and the multi-parametric assays were gathered in a singular dataset. Dimensionality of the data was reduced using a two-stage PCA-LDA algorithm, as Howland and Park (2004) have shown that using Principal Component Analysis (PCA) as a pre-processing step for Linear Discriminant Analysis (LDA) when performing dimensionality reduction allows for effective discrimination while successfully decreasing the dimensionality (7). Variance captured by each individual single-trait and multi-

parametric behavioral assay was calculated. PCA-LDA reduced the dimensionality of the data from the initial 89 dimensions to N-1 dimension, where N is the amount of classes, resulting in 7 linear discriminants. To identify the capability of these assays to differentiate the different mice models and sex- and environmental-modifiers, the SciKitLearn (v0.24.2) python library (8) was used to train linear classifiers. Because the hold-out approach, where the data is initially split between a training and test datasets for validation of the machine learning algorithm, creates larger uncertainties at smaller sample sizes (9), we selected the Leave One Out (LOO) cross-validation to ensure that the classifiers were properly fitted. In short, LOO is a method for which the final model is trained on the entire dataset, allowing for higher accuracy in smaller datasets (9), while still allowing for proper cross-validation through repeated linear classifier training within the dataset. This is done by taking multiple "folds" within the datasets as training datasets and taking the mean accuracy over these different validation folds as the cross-validation accuracy (9–11).

After cross-validation, a Linear Support Vector Machine (SVM) classification method was adopted to classify the behavioral data as the classifier was found to have the best cross-validation output. To further prevent overfitting, a regularization factor was used to impose a penalty on the complexity of the model; this allows simpler classification barriers to be more cost-efficient during training, thus allowing for more generalizable models as long as underfitting has not taken place (12). For the SVM, a regularization factor of $C = 0.1$ was utilized during training after hyperparameter tuning between 1.0 and 0.01, while taking order of magnitude steps, showed this value had the highest accuracy while not being overfitted according to the cross-validation metric.

Supplementary References

1. Serra I, Manusama OR, Kaiser FMP, Floriano II, Wahl L, van der Zalm C, *et al.* (2021): Activated PI3Kδ syndrome, an immunodeficiency disorder, leads to sensorimotor deficits recapitulated in a murine model. *Brain, Behavior, & Immunity - Health* 18: 100377.
2. Lopes G, Bonacchi N, Frazão J, Neto JP, Atallah BV, Soares S, *et al.* (2015): Bonsai: an event-based framework for processing and controlling data streams. *Front Neuroinform* 9:7.
3. Ben-Shaul Y (2017): OptiMouse: a comprehensive open source program for reliable detection and analysis of mouse body and nose positions. *BMC Biol* 15: 41.
4. Wahl L, Punt AM, Arbab T, Willuhn I, Elgersma Y, Badura A (2022): A Novel Automated Approach for Improving Standardization of the Marble Burying Test Enables Quantification of Burying Bouts and Activity Characteristics. *eNeuro* 9. https://doi.org/10.1523/ENEURO.0446-21.2022
5. Badura A, Verpeut JL, Metzger JW, Pereira TD, Pisano TJ, Deverett B, *et al.* (2018): Normal cognitive and social development require posterior cerebellar activity. *Elife* 7. https://doi.org/10.7554/eLife.36401
6. Verpeut JL, Bergeler S, Kislin M, William Townes F, Klibaite U, Dhanerawala ZM, *et al.* (2023): Cerebellar contributions to a brainwide network for flexible behavior in mice. *Commun Biol* 6: 605.
7. Howland P, Park H (2004): Equivalence of Several Two-stage Methods for Linear Discriminant Analysis. *Proceedings of the 2004 SIAM International Conference on Data Mining (SDM)*. Society for Industrial and Applied Mathematics, pp 69–77.
8. Pedregosa F, Varoquaux G, Gramfort A, Michel V, Thirion B, Grisel O, *et al.* (2012, January 2): Scikit-learn: Machine Learning in Python. *arXiv [cs.LG]*. Retrieved from

http://arxiv.org/abs/1201.0490

9. Eertink JJ, Heymans MW, Zwezerijnen GJC, Zijlstra JM, de Vet HCW, Boellaard R (2022): External validation: a simulation study to compare cross-validation versus holdout or external testing to assess the performance of clinical prediction models using PET data from DLBCL patients. *EJNMMI Res* 12: 58.

10. Fushiki T (2011): Estimation of prediction error by using K-fold cross-validation. *Stat Comput* 21: 137–146.

11. Yadav S, Shukla S (2016): Analysis of k-Fold Cross-Validation over Hold-Out Validation on Colossal Datasets for Quality Classification. *2016 IEEE 6th International Conference on Advanced Computing (IACC)* 78–83.

12. Tian Y, Zhang Y (2022): A comprehensive survey on regularization strategies in machine learning. *Inf Fusion* 80: 146–166.
